# Protein post-translational modifications in serine synthetic pathway: functions and molecular mechanisms

**DOI:** 10.1186/s12964-025-02327-4

**Published:** 2025-07-01

**Authors:** Mincong Shu, Yuhan Liu, Jianbin Wang

**Affiliations:** 1 The Fifth Clinical School of Hubei University of Medicine, Shiyan, 442000 China; 2 College of Medicine, Jinggangshan University, Ji’an, 343000 China; 3 Department of Thoracic Surgery, The First Affiliated Hospital of Nanchang University, Nanchang, 330006 China

**Keywords:** Serine synthetic pathway, Post-translational modification, PHGDH, PSAT1, PSPH

## Abstract

Serine is a non-essential amino acid, serving as a precursor for other amino acids, lipids, and nucleotide synthesis. Its supply is ensured by two main mechanisms: exogenous uptake and endogenous synthesis. The serine synthesis pathway (SSP) connects glycolysis with the one-carbon cycle and plays an important role in cellular homeostasis by regulating substance synthesis, redox homeostasis, and gene expression. The de novo SSP involves three successive enzymatic reactions catalyzed by phosphoglycerate dehydrogenase (PHGDH), phosphoserine aminotransferase 1 (PSAT1), and phosphoserine phosphatase (PSPH). Post-translational modifications (PTMs), as essential regulatory mechanisms of proteins, play pivotal roles in physiological and pathological processes. This review focuses on the regulatory mode of PTMs on PHGDH, PSAT1, and PSPH, including phosphorylation, ubiquitination, acetylation, methylation, S-palmitoylation, S-nitrosylation, deamidation, SUMOylation, and lactylation. We summarize how these PTMs participate in the metabolic reprogramming of SSP. It helps us better understand the molecular mechanisms and physiological significance of the PTM network in serine synthetic metabolism, providing guidance for subsequent research and development in the future.

## Background

**Fig. 1:**
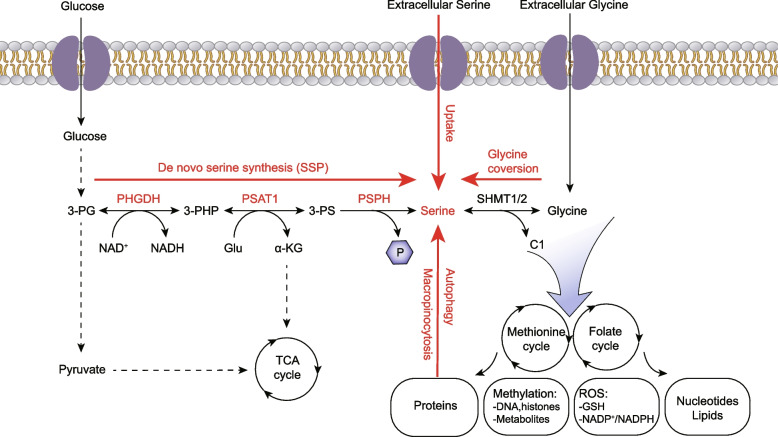
The sources and metabolic functions of serine. Serine can be taken up directly from the extracellular environment, derived from intracellular protein via autophagy, produced by glycine conversion, or synthesized de novo via the serine synthesis pathway, to supply a major source of energy and a critical cellular component. Abbreviations: SSP, Serine synthesis pathway; 3-PG, 3-phosphoglycerate; 3-PHP, 3-phosphohydroxypyruvate; 3-PS, 3-phosphoserine; PHGDH, phosphoglycerate dehydrogenase; PSAT1, phosphoserine aminotransferase 1; PSPH, phosphoserine phosphatase; SHMT1/2, serine hydroxymethyltransferase 1/2; NAD^+^, nicotinamide adenine dinucleotide; NADH, reduced nicotinamide adenine dinucleotide; Glu, glutamine; α-KG, α-ketoglutarate; TCA, tricarboxylic acid; ROS, reactive oxygen species; GSH, glutathione; NADP^+^, nicotinamide adenine dinucleotide phosphate; NADPH, reduced nicotinamide adenine dinucleotide phosphate; P, phosphate group

Amino acids are basic substances for sustaining life activities, extensively contributing to intracellular metabolic pathways and supporting the normal physiological functions [1]. Serine serves as a critical precursor in the synthesis of various cellular components. As one of the non-essential amino acids, serine is second only to glutamine in metabolic utilization by cancer cells, highlighting its biological significance [2, 3]. It donates one-carbon units and participates in the biosynthesis of proteins, nucleotides, lipids, and other macromolecules. Additionally, it maintains cellular redox homeostasis by increasing the production of antioxidant glutathione (GSH) and other reducing agents. Serine also regulates methylation reactions by supporting the generation of S-adenosylmethionine (SAM) in folate and methionine cycles [4]. Serine can be acquired through transporter-mediated extracellular uptake, intracellular proteolytic degradation, de novo synthesis, or conversion from glycine [5]. Under fasting conditions, approximately 70% of endogenous serine is derived from the serine synthesis pathway (SSP) by three successive enzymatic reactions [6] (Fig. 1). In the pathway, glycolysis intermediate 3-phosphoglycerate (3-PG) is oxidized to 3-phosphohydroxypyruvate (3-PHP) by phosphoglycerate dehydrogenase (PHGDH), with cofactor nicotinamide adenine dinucleotide (NAD^+^) reduced to reduced nicotinamide adenine dinucleotide (NADH). Subsequently, 3-PHP undergoes phosphoserine aminotransferase 1 (PSAT1)-mediated transamination with glutamate to form 3-phosphoserine (3-PS). In this process, glutamate is converted into α-ketoglutarate (α-KG). Finally, phosphoserine phosphatase (PSPH) catalyzes the dephosphorylation of 3-PS to serine. Moreover, serine can be reversibly converted to glycine and one-carbon units by serine hydroxymethyltransferase 1/2 (SHMT1/2) [7]. Serine modulates diverse biochemical reactions, and SSP alteration becomes a critical factor driving cancer progression, nervous system diseases, and metabolic syndrome.

**Fig. 2:**
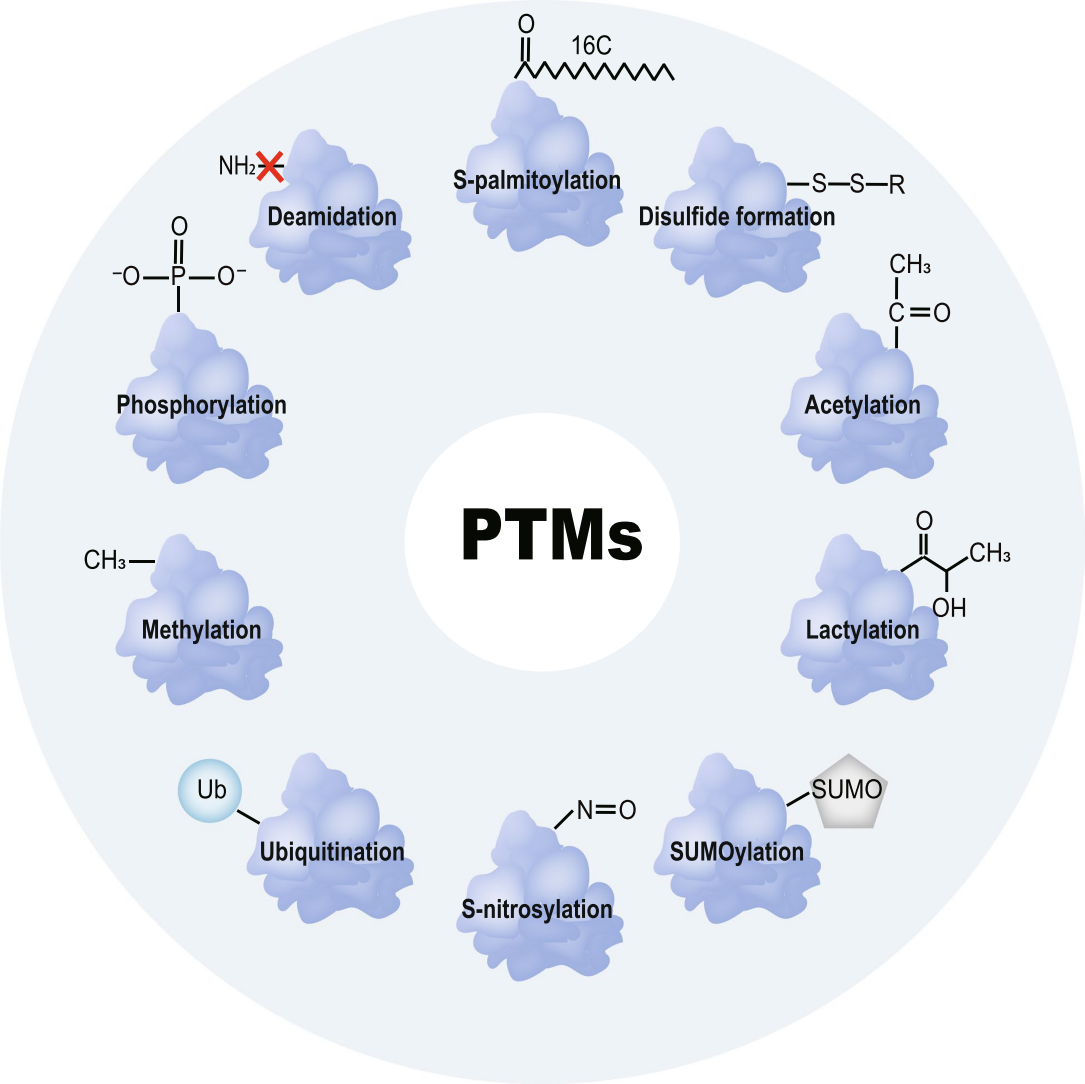
Schematic illustration of post-translational modifications associated with serine synthesis metabolism. The chemical structures of modifications include S-palmitoylation, acetylation, lactylation, SUMOylation, S-nitrosylation, ubiquitination, methylation, phosphorylation, deamidation, and disulfide formation. Abbreviations: PTMs, post-translational modifications; Ub, ubiquitin; SUMO, small ubiquitin-like modifier

The Human Genome Project revealed that the human proteome contains over one million distinct proteins and post-translational modifications (PTMs) significantly enhance the functional diversity of the proteome [8, 9]. PTMs are biochemical processes in which specific chemical groups are enzymatically added to or removed from specific amino acid residues. These dynamic processes precisely regulate protein conformation, activity, stability, localization, and interactions [10, 11]. Protein phosphorylation, the first identified PTM, was discovered in the early twentieth century [12]. Subsequently, the broader significance of PTMs began to be acknowledged. Over 600 PTMs are now known, including common modifications such as phosphorylation, acetylation, methylation, and ubiquitination. With the rapid advancement of mass spectrometry-based proteomics and associated detection technologies, several novel PTMs were found, such as succinylation and lactylation [8, 13–15]. Although single PTMs can significantly influence protein function, multiple PTMs are also intricately interconnected, collectively regulating cellular activities through antagonistic or synergistic mechanisms. Ultimately, PTMs regulate critical cellular biological functions involved in cell proliferation, immune response, signal transduction, and so on [16] (Fig. 2).

Approximately 10% of glycolytic intermediate 3-PG enters SSP for serine synthesis, undergoing a three-part enzymatic reaction mediated by PHGDH, PSAT1, and PSPH [17]. Here, we systematically review key PTMs linked to SSP, including phosphorylation, ubiquitination, acetylation, methylation, S-palmitoylation, redox modification, deamidation, SUMOylation, and lactylation. We summarize their modification characteristics, regulatory mechanisms, and functions in physiological and pathological states.

## Post-translational modifications of PHGDH

**Fig. 3:**
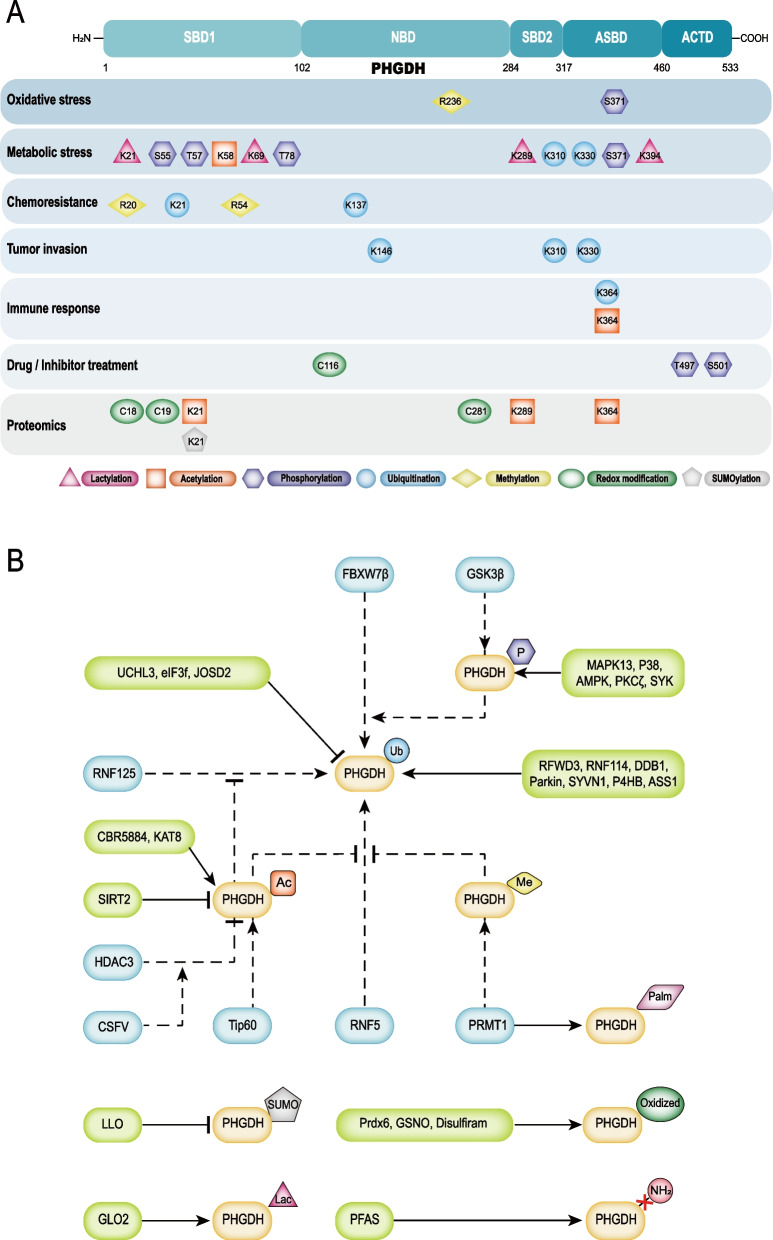
Overviews of post-translational modifications associated with PHGDH. **A** PHGDH is composed of SBD1/2, NBD, ASBD, and ACTD. In response to various types of stimuli, PHGDH undergoes a variety of post-translational modifications on specific amino acid residues. **B** Schematic representation of PTMs regulator mechanisms on PHGDH. The solid line represents the regulatory mechanism on PHGDH of individual PTMs. The dotted line represents the co-regulatory networks of multiple PTMs associated with PHGDH. Abbreviations: PHGDH, phosphoglycerate dehydrogenase; SBD, substrate-binding domain; NBD, nucleotide-binding domain; ASBD, allosteric substrate-binding domain; ACTD, aspartate kinase-chorismate mutase-TyrA domain; R, arginine; S, serine; K, lysine; T, threonine; C, cysteine. P, phosphate group; Me, methyl group; Ac, acetyl group; Ub, ubiquitin; SUMO, small ubiquitin-like modifier; Lac, lactyl group; Palm, palmitoyl group; FBXW7β, F-box and WD repeat domain containing 7 beta; GSK3β, glycogen synthase kinase 3 beta; RNF125, ring finger protein 125; HDAC3, histone deacetylase 3; CSFV, Classical swine fever virus; Tip60, tat interactive protein 60 kDa; RNF5, ring finger protein 5; PRMT1, protein arginine methyltransferase 1; UCHL3, ubiquitin carboxyl terminal hydrolase L3; eIF3f, eukaryotic translation initiation factor 3 subunit f; JOSD2, josephin domain-containing protein 2; MAPK13, mitogen-activated protein kinases 13; P38, mitogen-activated protein kinase 14; AMPK, AMP-activated protein kinase; PKCζ, protein kinase C zeta; SYK, spleen tyrosine kinase; RFWD3, ring finger and WD repeat domain 3; RNF114, ring finger protein 114; DDB1, damage specific DNA binding protein 1; SYVN1, synoviolin 1; P4HB, prolyl 4-hydroxylase subunit beta; ASS1, argininosuccinate synthase 1; KAT8, lysine acetyltransferase 8; SIRT2, sirtuin 2; LLO, listeriolysin O; Prdx6, peroxiredoxin 6; GSNO, S-nitrosoglutathione; GLO2, glyoxalase 2; PFAS, phosphoribosylformylglycinamidine synthase

PHGDH belongs to the D-isomer-specific 2-hydroxyacid dehydrogenase family. As an NAD^+^-dependent enzyme, PHGDH regulates SSP by catalyzing the rate-limiting conversion of 3-PG to 3-PHP [18]. PHGDH exists in three different subtypes, differing in domain composition, that have been referred to as subtypes I, II, and III. Human PHGDH (subtype I) comprises different domains: the substrate-binding domain (SBD), nucleotide-binding domain (NBD), allosteric substrate-binding domain (ASBD), and aspartate kinase-chorismate mutase-TyrA domain (ACTD) [19]. Subtype III is composed only of SBD and NBD. Due to the lack of ASBD and ACTD, the subunit interface occurs in the catalytic domains as a dimeric configuration. Compared with subtype III, subtype II contains an additional ACTD at the C-terminus. The most well-studied subtype II is *E. coli* PHGDH [20]. PHGDH is universally expressed in all organisms and overexpressed in various metabolic diseases, especially in cancer. In liver macrophages, PHGDH suppresses the activation of NF-κB and MAPK signaling to alleviate metabolic dysfunction-associated fatty liver disease [21]. Over-expression of PHGDH facilitates lung adenocarcinoma (LUAD) cells resistant to erlotinib treatment by regulating DNA damage repair and nucleotide metabolism [22]. Liver cancer progression is associated with elevated protein intensity and nuclear localization of PHGDH, which synergistically functions with cMYC to drive chemokine expression and reshape the immune microenvironment [23]. PHGDH also regulates nervous system diseases. It is specifically expressed in astrocytes, and PHGDH-mediated serine synthesis promotes inflammatory cytokine production to support neuroinflammation and neurotoxicity [24]. PHGDH overexpression promotes cell proliferation and migration by activating SSP to generate biomass, energy, and reductants [25, 26]. PHGDH can be regulated by a variety of upstream signals, ultimately affecting physiological function. Here, we summarize the effects of PTMs on PHGDH (Fig. 3andTable 1).

**Table 1:** Overviews of PTM classification and function associated with PHGDH

PTM Type	PTM Site	Enzyme/Molecule	Regulation of biological function	Reference
Phosphorylation	S371	MAPK13	liver injuries under oxidative stress	[31]
	S371	P38	tumor development under nutrient stress	[32]
	S55	AMPK		
	T57, T78	PKCζ	intestinal cancer development under nutritional stress	[34]
	T497, S501	GSK3β	colorectal cancer development	[35]
		SYK	antifungal immunity	[36]
Ubiquitination		FBXW7β	colorectal cancer development	[35]
		eIF3f		
	K137	RFWD3	chemoresistance of osteosarcoma	[40]
	K310, K330	RNF114	renal and hepatic steatosis	[41]
	K146	DDB1	cell malignancy and stemness of colorectal cancer, colorectal cancer metastasis	[42, 60]
	K330	Parkin	cell malignancy, stemness and chemoresistance of non-small cell lung cancer	[43–45]
		SYVN1	cell malignancy and aggressiveness of bladder cancer	[46]
		P4HB	cell apoptosis of skeletal muscle	[47]
		ASS1	triple-negative breast cancer development	[48]
	K310	UCHL3	cell malignancy and aggressiveness of colorectal cancer	[51]
		JOSD2	hepatocellular carcinoma development, cancer stem cell phenotype	[18, 52]
	K364	RNF125	antiviral immunity	[56]
	K21	RNF5	redox homeostasis, chemoresistance of triple-negative breast cancer	[49]
Acetylation	K289, K364, K21	CBR5884		[64]
	K364	KAT8, HDAC3	antiviral immunity	[56]
	K58	Tip60, SIRT2	redox homeostasis, breast cancer development	[49]
Methylation	R20, R54, R236	PRMT1	chemoresistance of triple-negative breast cancer, therapeutic vulnerability of hepatocellular carcinoma, redox homeostasis, hepatocellular carcinoma development	[50, 66, 67]
S-palmitoylation		PRMT1	chemoresistance of triple-negative breast cancer	[50]
Redox modification	C281, C18, C19	Prdx6		[73, 74]
	C116	GSNO		[75]
	C116	Disulfiram	tumor development	[76]
Deamidation		PFAS		[80]
SUMOylation	K21	LLO		[84]
Lactylation	K21, K69, K289, K394	GLO2	glycolytic flux	[88]

*Abbreviations: PTM* post-translational modification, *PHGDH* phosphoglycerate dehydrogenase, *MAPK13* mitogen-activated protein kinases 13, *P38* mitogen-activated protein kinase 14, *AMPK* AMP-activated protein kinase, *PKCζ* protein kinase C zeta, *GSK3β* glycogen synthase kinase 3 beta, *SYK* spleen tyrosine kinase, *FBXW7β* F-box and WD repeat domain containing 7 beta, *eIF3f* eukaryotic translation initiation factor 3 subunit f, *RFWD3* ring finger and WD repeat domain 3, *RNF114* ring finger protein 114, *DDB1* damage specific DNA binding protein 1, *SYVN1* synoviolin 1, *P4HB* prolyl 4-hydroxylase subunit beta, *ASS1* argininosuccinate synthase 1, *UCHL3* ubiquitin carboxyl terminal hydrolase L3, *JOSD2* josephin domain-containing protein 2, *RNF125* ring finger protein 125, *RNF5* ring finger protein 5, *KAT8* lysine acetyltransferase 8, *HDAC3* histone deacetylase 3, *Tip60* tat interactive protein 60 kDa, *SIRT2* sirtuin 2, *PRMT1* protein arginine methyltransferase 1, *Prdx6* peroxiredoxin 6, *GSNO* S-nitrosoglutathione, *PFAS* phosphoribosylformylglycinamidine synthase, *LLO* listeriolysin O, *GLO2* glyoxalase 2, *R* arginine, *S* serine, *K* lysine, *T* threonine, *C* cysteine

### Phosphorylation of PHGDH

More than 75% of proteins can be phosphorylated in eukaryotes [27]. Phosphorylation is an adenosine triphosphate (ATP)-dependent process in which a phosphate group covalently binds to a specific amino acid residue via the enzymatic action of protein kinases. Phosphorylated substrate proteins can reversibly lose phosphate groups through phosphatase activity [28, 29]. Phosphorylation acts as a molecular switch, regulating physiological processes such as enzymatic activity, protein stability, protein conformation, and protein–protein interaction [30].

During drug-induced liver injury, MAPK13 is activated. PHGDH is phosphorylated at S371 by MAPK13 upon oxidative stress, triggering its degradation via the chaperone-mediated autophagy pathway. The elimination of PHGDH attenuates SSP flux and GSH production, resulting in intracellular reactive oxygen species (ROS) accumulation. This redox imbalance enhances hepatocyte sensitivity to oxidative stress and exacerbates cholestatic liver injury. MAPK13 inhibition and dietary serine supplementation alleviate oxidative stress-associated liver injury [31].

Upon glucose deprivation, PHGDH-S371 is phosphorylated by P38, promoting its nuclear translocation. Meanwhile, AMP-activated protein kinase (AMPK) phosphorylates PHGDH at S55, selectively enhancing NADH production during malate-to-oxaloacetate oxidation. Phosphorylated PHGDH suppresses both NAD^+^ level and NAD^+^-dependent PARP1 activity, thereby inhibiting c-Jun transcriptional activity. Ultimately, nuclear PHGDH sustains cell proliferation in glucose-deprived liver pancreatic cancer cells [32].

Another study demonstrates that inhibiting PKCζ, an atypical protein kinase C subtype, enhances glutamine utilization via SSP during glucose deprivation. This metabolic adaptation upregulates PHGDH to sustain serine synthesis [33]. Mechanistically, PKCζ, a tumor suppressor, acts upstream of PHGDH to directly repress its expression under nutrient stress. PKCζ also phosphorylates PHGDH at T57 and T78, to inhibit its enzymatic activity by abrogating substrate binding. PHGDH inactivation reduces serine production from glutamine metabolism, impairing intestinal cancer cell adaptation to nutrient stress [34].

Glycogen synthase kinase-3 beta (GSK3β) phosphorylates PHGDH at its degron motif (T497/S501). The modification decreases PHGDH protein homeostasis through inducing FBXW7β‐mediated degradation. EGF or CHIR (GSK3 inhibitor) treatments stabilize PHGDH by inactivating GSK3β. Thus, EGF‐GSK3β‐FBXW7β axis regulates PHGDH expression, driving the Serine–Glycine–One–Carbon (SGOC) pathway to promote colorectal cancer progression [35].

Recent studies reveal that spleen tyrosine kinase (SYK) phosphorylates PHGDH through its catalytic activity. In immune cells, SYK overexpression activates PHGDH to balance SSP and redox homeostasis during fungal infections. *Aspergillus fumigatus* and *candida albicans* infection induce interaction between SYK and ACTD of PHGDH, upregulating PHGDH phosphorylation and shifting flux from glycolysis to SSP and one-carbon metabolism. Elevated serine, glycine, GSH/GSSG, and the reduced nicotinamide adenine dinucleotide phosphate (NADPH)/nicotinamide adenine dinucleotide phosphate (NADP^+^) ratio drive pro-inflammatory cytokines production, essential for antifungal immunity [36].

### Ubiquitination of PHGDH

Ubiquitination is a process in which one or more ubiquitin molecules are covalently linked to a substrate protein through a cascade of E1-E2-E3 enzymatic reactions [37]. Ubiquitination predominantly occurs at lysine residues, and ubiquitin can be conjugated via either the N-terminal methionine residue or one of seven lysine residues (K6, K11, K27, K29, K33, K48, and K63) [38]. Dysfunction of ubiquitination is intricately associated with numerous diseases.

#### Proteasome-dependent degradation

Ubiquitination is involved in approximately 80–90% of cellular protein degradation, and it is a necessary signal for activating proteasomal degradation of substrate proteins [39]. In this section, we will present an overview of PHGDH degradation in a ubiquitin–proteasome-dependent manner.

RFWD3, a RING-type E3 ligase, plays a crucial role in regulating cisplatin sensitivity in osteosarcoma. Mechanistically, RFWD3 interacts with the NBD of PHGDH, inducing K48-linked polyubiquitination at K137 and proteasome-mediated degradation. Cisplatin strengthens the RFWD3-PHGDH interaction, decreasing serine synthesis and elevating the NAD^+^/NADH ratio. This process activates nucleotide biosynthesis to support DNA repair and facilitate osteosarcoma chemoresistance [40].

Demethylase UTX recruits RNF114 to increase K48-linked ubiquitination of PHGDH at K310 and K330, leading to proteasomal degradation. Ultimately, the elimination of PHGDH suppresses circulating serine levels, triggering lipid accumulation and upregulating triglyceride synthesis genes. The UTX-PHGDH-serine axis regulates renal and hepatic steatosis under high-fat diet stress to impact metabolic homeostasis [41].

As an E3 ligase, DNA damage-binding protein 1 (DDB1) is a crucial component of the DDB1-CUL4 ubiquitin ligase complex. In colorectal cancer cells, the novel molecular glue, LXH-3–71, covalently binds to PHGDH-C281, promoting the interaction between DDB1 and PHGDH. DDB1 increases poly-ubiquitination and degradation of PHGDH, inhibiting colorectal cancer growth and stemness via an enzyme-independent mechanism. Therefore, specific therapeutic strategies targeting selective degradation offer substantial promise compared to enzymatic inhibitors [42].

Parkin, a tumor suppressor, ubiquitinates PHGDH at K330, with its IBR domain binding to PHGDH-SBD2. Their interaction reduces PHGDH protein levels through proteasomal degradation, suppressing serine synthesis and cell proliferation. Parkin knockout increases sensitivity to PHGDH inhibitors in breast and lung cancer cells [43, 44].

Further study confirms K330 as a key parkin-mediated ubiquitination site, making it susceptible to proteasomal degradation. Isocitrate dehydrogenase 1 (IDH1) counteracts this process by binding PHGDH-SBD2 to sterically hinder the interaction with parkin. The process suppresses the ubiquitination degradation of PHGDH. In non-small cell lung cancer (NSCLC), IDH1 regulates serine metabolism and redox homeostasis to maintain cancer stemness. Meanwhile, IDH1 knockdown sensitizes NSCLC to gemcitabine chemotherapy by impairing PHGDH-mediated pyrimidine synthesis [45].

A novel circular RNA (circSIRT5) is formed by back-splicing of SIRT5 exons 2–9. CircSIRT5 enhances the interaction between E3 ligase SYVN1 and PHGDH, promoting its ubiquitination and degradation. CircSIRT5/SYVN1/PHGDH complex promotes ferroptosis in bladder cancer, ultimately inhibiting malignant progression and invasion [46].

In skeletal muscle cells, PHGDH is involved in the mechanistic regulation of prolyl 4‐hydroxylase subunit beta (P4HB)-induced apoptosis. P4HB in extracellular vesicles, derived from oesophageal squamous cell carcinoma, induces muscle wasting via activating ubiquitin‐dependent degradation of substrate protein. P4HB overexpression accelerates PHGDH ubiquitination and degradation, reducing antiapoptotic protein Bcl‐2 stability and inducing apoptosis [47].

Argininosuccinate synthase (ASS1) acts as a tumor suppressor in triple-negative breast cancer (TNBC). ASS1 directly binds to PHGDH and enhances its ubiquitination and degradation. Elevated PHGDH restricts serine biosynthesis, while ASS1 knockdown partially rescues TNBC progression under serine deprivation [48].

RNF5 is an E3 ligase to ubiquitinates PHGDH, inducing its degradation and subsequently SSP inhibition. Meanwhile, PHGDH acetylation at K58 disrupts its association with RNF5, inhibiting proteasome-mediated degradation. RNF5 and PHGDH expressions inversely correlate in PR^−^ breast cancer, disrupting redox homeostasis to suppress breast tumorigenesis [49].

Protein arginine methyltransferase-1 (PRMT1) regulates the interplay between methylation and ubiquitination of PHGDH. Mechanistically, PRMT1 catalyzes the dimethylation of PHGDH at R20. RNF5, as an E3 ligase, binds to PHGDH through its SBD1, where R20 and K21 are located. RNF5-mediated polyubiquitination can decrease the stability of PHGDH. The unmethylable R20K-mutant induces higher polyubiquitination at the adjacent K21 of PHGDH. Consequently, the dimethylation of R20 competes with the polyubiquitination of K21 to stabilize PHGDH and promote its enzymatic activity, driving SSP and chemoresistance in TNBC [50].

A study mentioned in the previous section elucidates that PHGDH phosphorylation can modulate its ubiquitination. Mechanistically, FBXW7β, a potential E3 ligase of PHGDH, can recognize phosphorylated PHGDH and decrease its stability by the ubiquitin–proteasome pathway. In contrast, the groups of ubiquitinated substrate protein can be reversibly removed by deubiquitination. Eukaryotic translation initiation factor 3 subunit f (eIF3f) counteracts this process through deubiquitination, rescuing PHGDH from proteasomal degradation by removing K48-linked ubiquitin chains. The FBXW7β-eIF3f-PHGDH complex dynamically regulates PHGDH stability, influencing SAM levels and SGOC signaling to control cell growth and tumorigenicity [35].

Furthermore, we also summarize other regulatory mechanisms of PHGDH deubiquitination. The circMYBL2 is a circular RNA encoding a 185 amino acid protein (p185) in colorectal cancer. P185 binds the C1 domain of ubiquitin carboxyl terminal hydrolase L3 (UCHL3) to competitively impede its association with PHGDH. As a deubiquitinase, UCHL3 stabilizes PHGDH by removing K310 ubiquitination. Subsequently, circMYBL2-encoded p185 counteracts this stabilization, suppressing PHGDH-mediated serine synthesis to inhibit colorectal cancer progression and aggressiveness [51].

PHGDH is highly expressed in a LUAD subset with poor prognosis, regulated by the ubiquitin proteasome system. Josephin Domain-containing 2 (JOSD2) knockdown results in a PHGDH reduction of more than 80%. As the key deubiquitinase, it stabilizes PHGDH to activate serine-glycine-folate metabolism, supporting DNA/RNA biosynthesis for cell proliferation and migration [18]. Subsequent study has shown the same mechanism in hepatocellular carcinoma (HCC). JOSD2 depletion enhances the ubiquitination degradation of PHGDH, thereby inhibiting HCC proliferation and cancer stem cell (CSC) phenotype with prognostic and functional significance [52].

#### Proteasome-independent degradation and other functions

Ubiquitin–proteasome system and autophagy are two major cellular degradation machineries in eukaryotes [53]. K48-linked ubiquitination activates canonical proteasomal degradation. Nevertheless, K63-linked ubiquitination does not target the proteasome, but instead DNA damage repair, cell signaling, and selective autophagy [54, 55]. Classical swine fever virus (CSFV) inhibits serine metabolism-mediated innate immunity. Mechanistically, CSFV infection induces PHGDH deacetylation to expose the K364 site, enabling RNF125-mediated K63-linked ubiquitination at K364. Subsequently, PHGDH is targeted for autophagy-lysosome degradation by p62/NDP52. CSFV infection disrupts serine metabolism to impair antiviral immunity by regulating PHGDH expression [56].

Also, structurally distinct ubiquitin modifications are recognized by effector proteins, resulting in diverse functional outcomes, such as degradation, signal transduction, and alteration in subcellular localization [57]. mono-ubiquitination often alters the interaction, localization, and transport of protein substrates [58, 59]. DDB1 monoubiquitinates PHGDH at K146, enhancing enzymatic activity instead of stability by recruiting DNAJA1 to facilitate tetrameric formation. This modification increases serine and SAM levels, promoting SETD1A-mediated histone methylation of cell adhesion genes LAMC2/CYR61. Ultimately, DDB1-mediated PHGDH mono-ubiquitination drives colorectal cancer metastasis [60].

In conclusion, for the different regulatory mechanisms of PHGDH ubiquitination, there are multiple therapeutic potentials. The elimination of PHGDH induced by ubiquitin-proteasomal degradation can attenuate the stemness of colorectal cancer cells, and this phenomenon is not affected by the enzyme activity of PHGDH [42]. It reveals that selective ubiquitin-proteasomal degradation of PHGDH may be a more viable therapeutic strategy compared to PHGDH enzyme inhibitors. In NSCLC, ubiquitination affects the sensitivity to gemcitabine treatment by mediating PHGDH protein homeostasis [45], suggesting that PHGDH ubiquitination is a potential target, which induces low protein expression and low nucleotide biosynthesis, thereby weakening the resistance to chemotherapeutic drugs targeting DNA. For non-degradation pathways, PHGDH is monoubiquitinated to enhance its activity and promote the metabolite intermediate generation, thereby promoting colorectal cancer metastasis [60]. Therefore, therapeutic strategies targeting ubiquitination can also directly inhibit the enzymatic activity of PHGDH to regulate cell metabolism.

### Acetylation of PHGDH

Acetylation involves the enzymatic or non-enzymatic covalent attachment of acetyl groups to the amino acid residues [61]. Lysine acetylation is reversible, and its homeostasis is maintained by lysine acetyltransferases (KATs) and lysine deacetylases [62]. Based on cofactor specificity, deacetylases are classified into two categories: Zn^2+^-dependent histone deacetylases (HDAC1-11) and NAD^+^-dependent sirtuins (SIRT1-7) [63]. Disrupted acetylation homeostasis is implicated in functional impairments and disease progression.

Treatment with CBR5884, a PHGDH-specific inhibitor, restricts colon cancer cell proliferation. Proteomic analysis reveals that PHGDH may undergo acetylation modification at K289 and K364. After serine deprivation, the acetylation site at position K364 still exists, while the loss of position K289 produces a new acetylation site at K21. Acetylation modification plays an important role for PHGDH in response to serine deprivation. Meanwhile, ATF4 activates PHGDH transcription to subsequently increase its protein levels in colon cancer cells under serine deprivation. Therefore, exogenous restriction of serine utilization regulates both transcription and acetylation modification levels of PHGDH [64].

CSFV evades immune surveillance by altering serine metabolism. KAT8 can directly bind and acetylate PHGDH at K364 via acetyltransferase activity, while CSFV-NS4A recruits HDAC3 to deacetylate this site. HDAC3-mediated deacetylation triggers its lysosome degradation and impairs enzymatic activity by reducing NAD^+^ binding. Ultimately, CSFV infection induces PHGDH deacetylation to suppress serine biosynthesis, weakening host immunity to promote viral replication [56].

Tip60 interacts with and stabilizes PHGDH by K58 acetylation, blocking RNF5-mediated ubiquitination. SIRT2 overexpression reverses its acetylation at K58. Tip60 and SIRT2 cooperatively regulate PHGDH stability in response to glucose deprivation. Reduced PHGDH-K58 acetylation leads to serine metabolic dysfunction, ROS accumulation, and proliferation inhibition [49].

### Methylation of PHGDH

Protein methylation involves the enzymatic transfer of a methyl group from the methyl donor SAM to specific amino acid residues. It predominantly occurs on lysine and arginine residues, although histidine, asparagine, and glutamine can also be methylated [65]. Methylation occurs on both histones and non-histone proteins, regulating gene transcription and protein function.

Methyltransferase PRMT1 di-methylates R20 and mono-methylates R54 of PHGDH in chemoresistant TNBC. The mutation at R20K or R54K significantly reduces enzymatic activity. In paclitaxel-sensitive cells, PHGDH is unmethylated and undergoes polyubiquitination degradation. In paclitaxel-resistant cells, the R54me1 contributes to maintaining affinity for NAD^+^ and 3-PG, as a substrate or coenzyme of PHGDH. Meanwhile, R20me2 prevents polyubiquitination to stabilize the active enzyme. PRMT1 increases the methylation and enzymatic activity of PHGDH to catalyze the biotransformation of glucose into SSP, delivering substrates for cell survival [50].

In HCC, the mRNA level and protein level of PHGDH are down-regulated, whereas serine synthesis is increased. PRMT1 mono-methylates PHGDH at R236 (located at the catalytic center), enhancing the substrate 3-PG affinity. PRMT1-mediated R236me1 and activation of PHGDH promote serine metabolism to sustain redox homeostasis and HCC progression, representing a therapeutic vulnerability [66].

A recent study confirms the methylation function of PRMT1 on PHGDH. In HCC, E3 ubiquitin FBXO7 ubiquitinates PRMT1 at K37, inducing its proteasomal degradation. PRMT1-mediated R236me1 of PHGDH promotes serine synthesis and ameliorates redox stress. PRMT1 downregulation, mediated by FBXO7, can repress PHGDH catalytic activity to decrease SSP and decrease antioxidant production. FBXO7–PRMT1–PHGDH axis regulates redox homeostasis and tumor survival [67].

### S-palmitoylation of PHGDH

Palmitoylation is a lipid-mediated PTM where palmitoyl groups, C16 fatty acid chains, are covalently attached to substrate protein residues via palmitoyl acyltransferases (PATs) [68]. Based on the linkage type, protein palmitoylation is categorized into three categories: S-, N-, and O-palmitoylation [69]. Palmitoylation is reversible, with depalmitoylases removing palmitoyl groups from cysteine residues. This dynamic process influences diverse cellular functions, including protein stability, subcellular localization, membrane transport, enzymatic activity, and signal transduction [68, 70]. PRMT1 regulates S-palmitoylation of PHGDH in paclitaxel-resistant TNBC. PRMT1-mediated methylation activates PHGDH to drive serine synthesis and α-KG production, which is converted to citrate and acetyl-CoA for fatty acid synthesis. In addition, PRMT1-mediated PHGDH activation also stabilizes fatty acid synthase (FASN) to enhance de novo fatty acid synthesis. Palmitate, generated from fatty acids, induces S-palmitoylation of PHGDH and FASN, creating a positive feedback loop to amplify fatty acid synthesis. PHGDH S-palmitoylation is a potential therapeutic target for overcoming chemoresistance [50].

### Redox modification of PHGDH

Cysteine thiol redox modifications are primarily mediated by ROS, reactive sulfur species, and reactive nitrogen species, representing a key regulatory mechanism for cysteine function [8]. S-nitrosylation, a reversible and predominantly non-enzymatic process, occurs in diverse functional proteins. It mediates nitric oxide’s biological effects in cellular signaling and acts as a redox-dependent modulator of physiological functions [71]. Disulfide formation, a prevalent reversible cysteine modification, determines protein structure, catalytic activity, and signal transduction processes [72].

Peroxiredoxin 6 (Prdx6), a member of the 1-Cys peroxiredoxin subfamily, alters redox homeostasis. In siRNA-Prdx6-treated hepatoblastoma cells, metabolomic analysis reveals that serine, 3-PG, and GSH levels are increased, indicating the diversion from the glycolytic pathway toward SSP. PHGDH contains several redox-sensitive cysteine residues in highly reduced states. Redox proteomics identified redox changes at PHGDH C281 and the peptide containing adjacent C18/C19. C281 is close to the essential active site H293, and C18/C19 are close to K21, a site for acetylation. Reversible redox changes of PHGDH could affect its catalytic and regulatory properties, resulting in metabolic reprogramming [73].

Additional mass spectrometry-based methodology identified the redox-sensitive C281 of PHGDH. Cysteine S-nitrosylation affects protein enzymatic activity, localization, and stability. Proteomic studies show that enzymes regulating glycolysis, oxidative phosphorylation, and amino acid metabolism are frequently S-nitrosylated. In total, 1,011 S-nitrosocysteine residues across 647 proteins in mouse tissues have been identified as targets of S-nitrosylation. PHGDH is modified by S-nitrosylation at C281 in wild-type mouse brain and thymus, dependent on endothelial nitric oxide synthase activity [74].

The combined proteomics and metabolomics suggest that the activity of at least 21 metabolic enzymes may be modulated by S-nitrosation. The small-molecule S-nitroso-glutathione (GSNO) acts as a transnitrosation donor. GSNO treatment increases PHGDH S-nitrosation and inhibits its enzymatic activity. C116, a critical cysteine residue for PHGDH activity, regulates structural integrity. S-nitrosation of C116 either disrupts PHGDH dimerization or induces an S-nitrosation-mediated disulfide formation, resulting in deactivation [75].

Disulfiram, an aldehyde dehydrogenase inhibitor used as a treatment for chronic alcoholism, is also a potent PHGDH inhibitor through a covalent allosteric mechanism [19, 76, 77]. Mechanistically, disulfiram oxidizes PHGDH at C116, forming a disulfide bridge with the adjacent monomer. The specific cysteine oxidation disrupts the active tetramer into inactive dimers or a lesser inactive monomer intermediate of PHGDH. Disulfiram suppresses SSP by altering oligomerization, ultimately restricting bladder cancer cell proliferation. PHGDH inhibition by disulfiram contributes to its overall anticancer activity [76].

### Deamidation of PHGDH

Deamidation is defined as the removal of ammonia from the amide group of asparagine or glutamine residues through hydrolysis, resulting in the formation of aspartic acid or glutamic acid. Deamidation can alter the charge properties and steric conformation of proteins to influence their function and stability [78, 79]. Phosphoribosylformylglycinamidine synthase (PFAS), as a deamidase, directly combines with PHGDH. Since deamidation at neutral pH can introduce a negative charge at the deamidation site, IPG strips are used for isoelectric focusing, and the charge states of PHGDH are assessed by two-dimensional gel electrophoresis. The presence of PFAS renders PHGDH more negatively charged and makes the isoelectric point of PHGDH shift to the positive pole, indicating that PHGDH is deamidated. However, tandem mass spectrometry analysis was not used to further specify the extent and the sites of deamidation. Ultimately, the deamidase activity of PFAS may promote tumor pathogenesis through PHDGH-mediated nucleotide supply [80].

### SUMOylation of PHGDH

In SUMOylation, a small ubiquitin-like modifier (SUMO) is linked to the ε-amino group of the lysine residues in the target protein via isopeptide bonds [81]. Five SUMO isoforms have been identified in eukaryotes, with SUMO1-3 being the predominant forms ubiquitously expressed in all cell types and organs [81, 82]. SUMOylation regulates protein stability, localization, and activity, with a primary focus on intranuclear processes [83]. The combination of quantitative proteomics and peptide immunocapture identified 295 SUMO1 and 167 SUMO2 modification sites on endogenous substrates in human cells. 130 sites are found both with SUMO1 and SUMO2, including PHGDH. Concurrently, high-throughput quantification of SUMOylation is analyzed upon a bacterial toxin listeriolysin O (LLO) treatment. In response to an external stimulus, the SUMOylation level of PHGDH at K21 is down-regulated in control versus LLO-treated cells. These methods map SUMOylation sites and quantify deSUMOylation of PHGDH under stimuli [84].

### Lactylation of PHGDH

Lactate serves as a substrate for lysine lactylation, with lactyl groups primarily derived from L-lactate or D-lactate. Elevated lactate production promotes lactylation formation [85]. Lactylation modulates gene transcription, signal transduction, enzymatic activity, protein folding, and structural stability [86, 87]. Glyoxalase 2 (Glo2) catalyzes methylglyoxal detoxification to D-lactate via lactylglutathione (LGSH), an intermediate in the glyoxalase system. Glo2 disruption leads to LGSH accumulation, driving non-enzymatic lysine lactylation of PHGDH. The lactylation levels at K21, K69, K289, and K394 are up-regulated, which inhibits PHGDH enzymatic activity, blocking 3-PG conversion to serine and arresting intracellular serine synthesis [88].

## Post-translational modifications of PSAT1

**Fig. 4:**
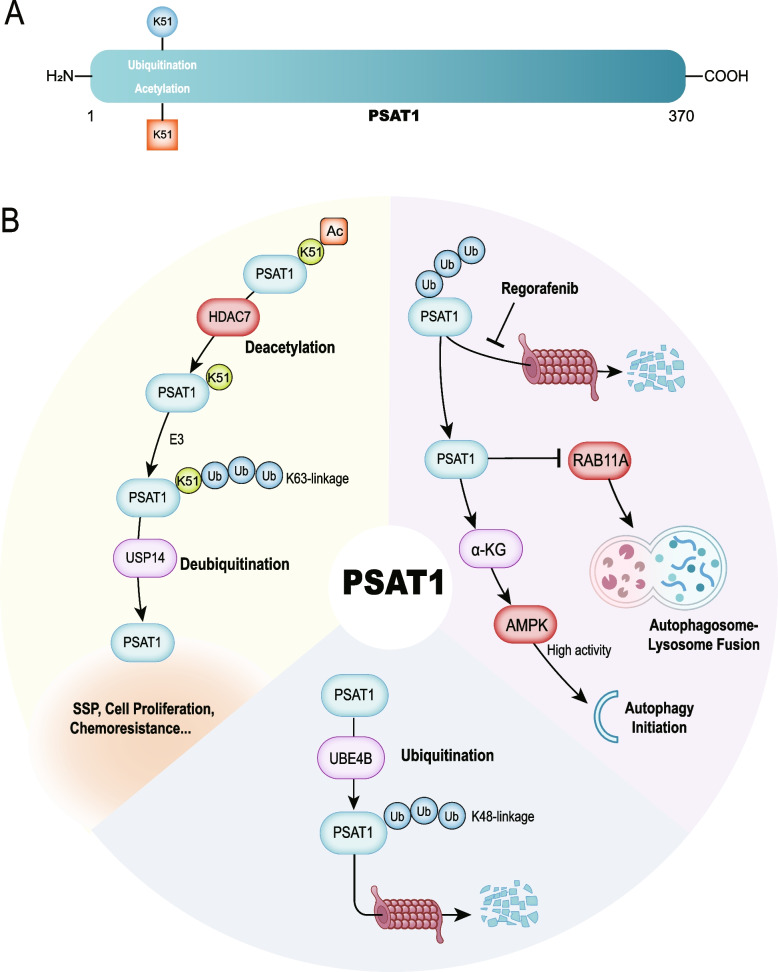
Overviews of post-translational modifications associated with PSAT1. **A** K51 is reported and confirmed as the ubiquitination and acetylation site of PSAT1. **B** Regorafenib and modification enzymes HDAC7, USP14, UBE4B regulate post-translational modifications of PSAT1. Abbreviations: PSAT1, phosphoserine aminotransferase 1; HDAC7, histone deacetylase 7; USP14, ubiquitin-specific protease 14; SSP, Serine synthesis pathway; UBE4B, ubiquitination factor E4B; RAB11A, Ras-related protein in brain 11 A; α-KG, α-ketoglutarate; AMPK, AMP-activated protein kinase; Ac, acetyl group; Ub, ubiquitin; K, lysine

PSAT1 belongs to the class V pyridoxal phosphate-dependent transaminase family. As the second key enzymes in SSP, it plays a central role in serine, glutamine, and glucose metabolism [89]. PSAT1 catalyzes the reversible conversion of 3-PHP to 3-PS with concomitant conversion of glutamate to α-KG. Thus, PSAT1 regulates serine metabolism by modulating functional metabolites, redox homeostasis, and methylation processes [7]. Its dysregulation is implicated in the occurrence and development of diseases. Vitamin D3 inhibits PSAT1 expression to attenuate the proliferation and differentiation of pulmonary fibroblasts via the MAPK pathway, relieving idiopathic pulmonary fibrosis [90]. Over-expression of PSAT1 generates α-KG and glutamine through serine metabolism, promoting muscle stem cell activation and reversing the aging-induced decline in muscle regeneration [91]. PSAT1 is upregulated in metastatic breast cancer and it can activate Wnt/β-catenin and Notch signaling pathways to facilitate cell stemness and aggressiveness [92]. In LUAD, aberrant upregulation of PSAT1 confers erlotinib resistance by suppressing apoptosis induced by ROS dependence and promotes tumor metastasis independently of its enzymatic activity [93].. PSAT1 plays an important role in the development of diseases. Here, we summarize the effects of PTMs on PSAT1 (Fig. 4).

### Ubiquitination of PSAT1

Regorafenib, an oral multi-kinase inhibitor, directly binds to PSAT1 and reduces its ubiquitination for protein stabilization. Elevated PSAT1 increases α-KG production, activating AMPK signaling, which is the upstream signal pathway of autophagy. Meanwhile, PSAT1 inhibits Ras-related protein in brain 11 A (RAB11A) expression, an autophagosome-lysosome fusion-associated protein, blocking autolysosome formation. Ultimately, regorafenib-mediated PSAT1 stabilization contributes to autophagy initiation and lethal autophagy arrest, inducing glioblastoma suppression [94].

The proliferation and tumorigenicity of LUAD cells are dependent on PSAT1 expression. UBE4B, the E3 ligase for PSAT1, increases K48-linked ubiquitination to trigger proteasomal degradation. In addition, K63-linked ubiquitination at PSAT1-K51 recruits deubiquitinase ubiquitin-specific protease 14 (USP14), decreasing ubiquitination level to escape degradation. Ultimately, elevated PSAT1 promotes serine metabolism and LUAD cell proliferation [95].

### Acetylation of PSAT1

In LUAD, PSAT1 acetylation increases its ubiquitination to reduce protein stability. Mechanistically, HDAC7 is identified as a deacetylase of PSAT1 and deacetylates PSAT1 at K51. The acetylation impacts the association with deubiquitinase or E3 ligase of PSAT1. Collectively, HDAC7-mediated deacetylation stabilizes PSAT1, affecting serine metabolism and sensitivity of LUAD cells to chemotherapeutic drug cisplatin [95].

## Post-translational modifications of PSPH

**Fig. 5:**
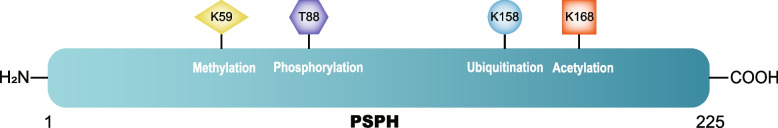
Overviews of post-translational modifications associated with PSPH. Some potential modification sites of PSPH are predicted by mass spectrometry-based proteomics. Abbreviations: PSPH, phosphoserine phosphatase; K, lysine; T, threonine

PSPH, a member of the phosphotransferase subfamily, is a protease characterized by a conserved N-terminal DXDXT motif. It is widely expressed in multiple tissues, including the heart, lung, brain, kidney, and skeletal muscle [96, 97]. PSPH catalyzes the irreversible dephosphorylation of SSP, converting 3-PS into serine. It regulates various physiological functions, and its alterations linked to serine metabolism disorders may drive the occurrence and development of multiple diseases [98]. PSPH expression was upregulated in both HCC tissues and cell lines. It can promote HCC cell proliferation and invasion via the AMPK/mTOR/ULK1 signaling pathway [99]. Meanwhile, PSPH also promotes serine metabolism to generate SAM and GSH. The accumulation of these metabolites facilitates monocytes/macrophages infiltration and inhibits CD8^+^ T lymphocytes recruitment, inducing HCC immune escape [100]. Over-expression of PSPH increases the activity of CDK2, a positive regulator of the cell cycle, facilitating cardiomyocyte proliferation and myocardial repair [101]. PSPH is also involved in the regulation of epileptic seizures by regulating astrocyte-derived serine levels [102]. However, the research on PTMs of PSPH is currently limited. So far, there are no verified modification sites and the specific PTM regulatory mechanisms of PSPH. Therefore, based on most of the reported mass spectrometry and modification omics, we summarize the potential PTM sites of PSPH, which will point out the future research direction of PSPH (Fig. 5).

In 2015, Tsai et al. quantified phosphorylation stoichiometry in gefitinib-sensitive (PC9) and resistant (PC9/gef) cells, identifying 7,392 phosphorylation sites. T88 was identified as a MAPK-mediated phosphorylation site of PSPH in PC9/gef cells [103].

In 2011, Kim et al. systematically analyzed ubiquitinated proteomes. In colorectal cancer cells, they identified 10,634 ubiquitination sites across 3,662 proteins, including the PSPH-K158 [104]. In 2018, Akimov et al. developed a monoclonal antibody UbiSite to recognize ubiquitin remnants after LysC digestion. 63,455 unique ubiquitination sites on 9,207 distinct proteins were identified. K158 was again identified as a ubiquitination site of PSPH [105].

In 2014, Lundby et al. provided an organ-wide atlas of lysine acetylation sites from different rat tissues analyzed by tandem mass spectrometry. A total of 16 organs from Sprague–Dawley albino rats revealed 15,474 lysine acetylation sites in 4,541 proteins. Among them, K168 was the acetylation site in PSPH from stomach tissue [106].

In 2016, Olsen et al. performed large-scale lysine mono-methylation profiling. They discovered 1,032 Kme1 sites in esophageal squamous cell carcinoma cells and an additional 1,861 sites in SMYD2-overexpressing cells, comprising the K59me1 site of PSPH [107].

In summary, PSPH may undergo phosphorylation, ubiquitination, acetylation, and methylation. Also, the corresponding potential sites have been predicted in these mass spectrometry results. Just like PHGDH and PSAT1, PTMs may also regulate the protein stability or enzymatic activity of PSPH, thereby affecting its downstream metabolic pathways. The specific mechanism requires further research. Overall, PTMs critically regulate SSP enzymes.

## Post-translational modifications of other proteins associated with SSP

Recent studies highlight the functional roles of PTMs in serine metabolism. In the previous section, we summarized the PTM states and direct regulatory effects of three key enzymes in the de novo SSP. However, in addition to PHGDH, PSAT1, and PSPH, the PTMs of other upstream proteins also affect the serine synthetic flux and regulate serine synthesis metabolism. Therefore, we will present an overview of PTMs'function on other proteins associated with SSP. Based on the regulatory pathway, we divide these modified proteins into two major categories: indirectly affecting serine synthesis metabolism by influencing the gene expression or enzymatic activity of SSP enzymes, or not involving SSP enzymes.

### Regulatory mechanisms of PTMs involving SSP enzymes

Serine deprivation rapidly induces ROS-mediated AMPK phosphorylation at T172 and AMPK-dependent HIF-1α stabilization. Activated HIF-1α binds to the promoter regions and enhances the transcriptional expression of SSP genes, thereby enhancing glucose-derived SSP to promote glioblastoma growth [108].

In leukemia-initiating cells, the calcium flux via ATP/P2X7 signaling promotes the phosphorylation of cyclic AMP response element-binding protein (CREB). CREB activates the expression of PHGDH through its transcriptional activity to maintain serine metabolism, enhancing the cell self-renewal capacities and leukemogenesis [109].

In HCC, USP10 can activate liver kinase B1 (LKB1) through deubiquitination. The LKB1/mTOR/ATF4 axis is an upstream regulatory pathway to control SSP via affecting the transcription of SSP enzymes. Loss of USP10 may increase serine biosynthesis and promote cell proliferation [110].

In colorectal cancer, cMYC is a transcriptional activator of PHGDH. eIF3f can deubiquitinate and stabilize cMYC, thereby increasing cMYC‐mediated PHGDH transcription and promoting SSP to facilitate colorectal cancer development [35].

In LUAD, histone acetyltransferase GCN5 is recruited to H3K27 on the PHGDH promoter region, facilitating its histone acetylation to activate PHGDH transcription, thereby promoting SSP and cell proliferation [111].

cMYC can be stabilized by the deacetylation activity of SIRT2. SIRT2/cMYC pathway reduces oxidative stress-induced apoptosis and promotes cholangiocarcinoma cell proliferation by transcriptionally promoting the downstream SSP pathway [112].

In cholangiocarcinoma, the combination of BET degradation and mTOR inhibition synergistically reduces acetylation of PSAT1-H3K27, inducing PSAT1 downregulation and subsequent SGOC pathway dysfunction [113].

In HCC cells, overexpression of GAPDH increases the H3K9 methylation levels of PHGDH and transcriptionally activates PHGDH to redirect glycolysis toward serine biosynthesis, consequently accelerating HCC development [114].

Serine deprivation induces methyltransferase G9A-mediated H3K9me1, maintaining transcriptional activation of SSP enzymes, thereby increasing serine and its downstream metabolite synthesis to sustain cell survival and proliferation in a wide range of cancer cell lines [115].

Ailanthone triggers autophagic degradation of methyltransferase KMT2A, which induces reduced H3K9me1 on the promoter region of PHGDH. Ailanthone epigenetically inhibits H3K9me1-mediated PHGDH transcription and suppresses lung metastasis of osteosarcoma via SSP downregulation [116].

Serine starvation increases SUMOylation level of nuclear erythroid-related factor-2 (NRF2) at K110. NRF2 is a pivotal transcription factor of PHGDH, and its SUMOylation promotes SSP by transcriptionally upregulating PHGDH, maintaining HCC tumorigenesis in response to metabolic stress [117].

In lenvatinib‐resistant HCC, glycolysis-driven lactate accumulation induces insulin-like growth factor 2 mRNA-binding protein 3 (IGF2BP3) lactylation, transcriptionally maintaining elevated NRF2 and PHDGH levels to activate SSP and strengthen the antioxidant capacity [118].

### Regulatory mechanisms of PTMs excluding SSP enzymes

When bisphosphoglycerate mutase (BPGM) is knocked out, phosphoglycerate mutase (PGAM1) phosphorylation and activity are decreased, leading to 3-PG accumulation. Due to the elevated glucose-serine flux, BPGM knockout cells show elevated production of both phosphoserine and serine [119].

In breast cancer stem cells, a novel hypoxia-specific circSTT3A directly interacts with HSP70, facilitating the recruitment of PGK1, which reduces its ubiquitination to increase protein stability. Upregulated PGK1 increases 3-PG accumulation and serine synthesis [120].

Insulin facilitates the deacetylase activity of HDAC3 and promotes the interaction between HDAC3 and phosphoglycerate kinase 1 (PGK1). HDAC3 promotes K220 deacetylation of PGK1, leading to elevated production of 3-PG and altering glucose metabolism and serine biosynthetic flux [121].

Pyruvate kinase M2 (PKM2) is arginine-methylated by methyltransferase CARM1 at R445 and R447. Its methylation enhances activity by triggering tetramer formation and further suppresses glucose flux toward serine synthesis in mouse embryonic fibroblasts and human breast cancer cells [122].

## Conclusions

Among all high-consumption nutrients, serine serves as the primary donor in folate and methionine cycles. Glycolytic intermediate 3-PG is converted to serine through a three-step enzymatic cascade, generating metabolite intermediates for lipid, nucleic acid, protein, and antioxidant biosynthesis. Currently, the transcriptional regulation mechanisms of PHGDH, PSAT1, and PSPH are relatively well-understood. Their transcriptional upregulation is driven by cMYC, ATF4, NRF2, YY2, and FOXC1 [44, 123, 124]. Compared to transcriptional control, protein PTMs have the advantage of rapid regulation. The fast development of mass spectrometry and the establishment of large-scale databases provide a necessary basis for PTM research. Studying PTMs on SSP-related proteins enhances understanding of serine metabolism regulation. In this review, we systematically analyzed the PTM-mediated regulatory mechanism of SSP and its impact on three key enzymes in this pathway. We found that PTMs can modulate the stability, interactions, structure, or activity of SSP-associated proteins, ultimately impacting downstream macromolecular synthesis, redox homeostasis, and other signaling pathways.

However, research on PTMs in serine synthesis metabolism remains preliminary, with some unresolved issues. (1) Current studies on the modification regulation of PHGDH are relatively comprehensive. But for the other two enzymes in SSP, especially PSPH, their regulatory mechanisms remain underexplored, providing significant research potential. (2) Several studies have proposed special compounds focusing on PTMs rather than the enzymatic activity of PHGDH. The molecular glue LXH-3–71 attenuates the cell stemness by inducing ubiquitin–proteasome degradation [42], and small molecule inhibitor disulfiram restricts cell proliferation by inducing cysteine oxidation-mediated oligomerization [76]. Other potential therapeutic targets require further exploration, remaining far from clinical translation. (3) Emerging techniques continue to discover novel modifications and crosstalk between different modifications, highlighting untapped research avenues. In the future, there are more potential prospects for exploring the mechanism of PTMs of serine synthetic metabolism. In summary, exploring the function of PTMs on SSP will contribute to a deeper understanding of intracellular metabolic regulation under physiological and pathological conditions, providing foundations for innovative therapeutic strategies and diagnostic biomarkers.

## Data Availability

No datasets were generated or analysed during the current study.

## Abbreviations

SSP: Serine synthesis pathway
PTM: Post-translational modification
PHGDH: Phosphoglycerate dehydrogenase
PSAT1: Phosphoserine aminotransferase 1
PSPH: Phosphoserine phosphatase
GSH: Glutathione
SAM: S-adenosylmethionine
3-PG: 3-Phosphoglycerate
3-PHP: 3-Phosphohydroxypyruvate
NAD^+^: Nicotinamide adenine dinucleotide
NADH: Reduced nicotinamide adenine dinucleotide
3-PS: 3-Phosphoserine
α-KG: α-Ketoglutarate
PSPH: Phosphoserine phosphatase
SHMT1/2: Serine hydroxymethyltransferase 1/2
SBD: Substrate-binding domain
NBD: Nucleotide-binding domain
ASBD: Allosteric substrate-binding domain
ACTD: Aspartate kinase-chorismate mutase-TyrA domain
ATP: Adenosine triphosphate
ROS: Reactive oxygen species
AMPK: AMP-activated protein kinase
GSK3β: Glycogen synthase kinase-3 beta
GSK3: Inhibitor CHIR
SGOC: Serine–Glycine–One–Carbon
SYK: Spleen tyrosine kinase
NADP^+^: Nicotinamide adenine dinucleotide phosphate
NADPH: Reduced nicotinamide adenine dinucleotide phosphate
eIF3f: Eukaryotic translation initiation factor 3 subunit f
DDB1: DNA damage-binding protein 1
IDH1: Isocitrate dehydrogenase 1
NSCLC: Non-small cell lung cancer
P4HB: Prolyl 4‐hydroxylase subunit beta
ASS1: Argininosuccinate synthase
TNBC: Triple-negative breast cancer
UCHL3: Ubiquitin carboxyl terminal hydrolase L3
HCC: Hepatocellular carcinoma
CSC: Cancer stem cell
LUAD: Lung adenocarcinoma
JOSD2: Josephin Domain-containing 2
CSFV: Classical swine fever virus
PRMT1: Protein arginine methyltransferase-1
KATs: Lysine acetyltransferases
HDAC: Histone deacetylases
SIRT: NAD^+^-dependent sirtuins
PATs: Palmitoyl acyltransferases
FASN: Fatty acid synthase
Prdx6: Peroxiredoxin 6
GSNO: S-nitroso-glutathione
PFAS: Phosphoribosylformylglycinamidine synthase
SUMO: Small ubiquitin-like modifier
LLO: Listeriolysin O
Glo2: Glyoxalase 2
LGSH: Lactylglutathione
RAB11A: Ras-related protein in brain 11A
UBE4B: Ubiquitination factor E4B
USP14: Ubiquitin-specific protease 14
Kme1: Mono-methyllysine
BPGM: Bisphosphoglycerate mutase
PGAM1: Phosphoglycerate mutase
DRP1: Dynamin-related protein 1
CREB: Cyclic AMP response element-binding protein
LKB1: Liver kinase B1
PGK1: Phosphoglycerate kinase 1
PKM2: Pyruvate kinase M2
SK1: Sphingosine kinase 1
PIMT: Protein L-IsoAsp methyltransferase
NRF2: Nuclear erythroid-related factor-2
IGF2BP3: Insulin-like growth factor 2 mRNA-binding protein 3
